# Knowledge about head and neck cancer among adolescents^☆^^☆☆^

**DOI:** 10.1016/j.bjorl.2025.101608

**Published:** 2025-06-04

**Authors:** Vinicius M. Vasconcellos, Leandro Luongo Matos, Flavio Carneiro Hojaij, Edevaldo Miguel Alves, Beatriz Costa N. Martins, Arthur C. Nava

**Affiliations:** a American School of Recife EAR, Recife, PE, Brazil; b Hospital Israelita Albert Einstein, Escola de Medicina, São Paulo, SP, Brazil; c Universidade de São Paulo (USP), Faculdade de Medicina (FM), São Paulo, SP, Brazil; d Universidade Federal de Pernambuco (UFPE), Recife, PE, Brazil; e Universidade Estadual de Pernambuco (UFPE), Recife, PE, Brazil

**Keywords:** Adolescent behavior, Health knowledge, Head and neck neoplasms

## Abstract

**Objective:**

To assess knowledge about head and neck cancer among adolescents from private schools, aged between 13 and22 years.

**Methods:**

200 questionnaires were applied among students, about the profile of adolescents and their knowledge about head and neck neoplasms, treatment, and teaching in schools on this topic. The results were analyzed both quantitatively and qualitatively.

**Results:**

Only 55 students (29.9%) identified HPV as a risk factor for the disease. 137 students (74.5%) identified the brain as an organ treated by head and neck surgery. As for the therapeutic strategies used for the treatment of neoplasms in the region, only 67 students (36.4%) are aware. On the topic of teaching in schools, 153 students (83.1%) relate to some deficiency in the approach and learning.

**Conclusion:**

Knowledge about head and neck cancer is insufficient. It is necessary to introduce in the curriculum basic cancer education in biology classes. The expansion of acquirements about cancer can provide better care and the ability to identify risk factors, initial signs, and symptoms of the disease, as well as the incorporation of healthy habits in families because these students can propagate valid information in their environment.

**Level of evidence:**

Level IV.

## Highlights

• Knowledge about head and neck cancer is lacking.
• It is necessary to introduce in the curriculum basic cancer education in biology classes.
• The expansion of acquirements about cancer can provide better care and the ability to identify risk factors, initial signs, and symptoms of the disease, as well as the incorporation of healthy habits in families because these students can propagate valid information in their environment.

## Introduction

Adolescence is a time of life marked by physical, behavioral, emotional, and cognitive changes. According to the World Health Organization (WHO), adolescence begins at the age of 10 and goes up to 19 years, however recent studies indicate that the concept of adolescence must be modified and includes age groups between 10 and 24 years.^1^ This period is characterized by new discoveries, anxieties, and conflicts, and the central point in this transition between childhood and adulthood is the definition of the new role that adolescents have in their social life.^2^, ^3^

According to data from the Ministry of Education (MEC) on the Basic Education Assessment System (Saeb), in 2017, carried out by the National Institute of Educational Studies and Research Anísio Teixeira (Inep), Brazilian secondary education has contributed minimally to technical knowledge to students.^4^ This technical approach to content, instead of a practical view and demonstration of the importance of knowledge, as well as the purpose of various disciplines, is one of the great questions during high school.^5^, ^6^

Even in adolescence, new habits and behaviors are incorporated, such as the use of tobacco and alcohol, unprotected sex, and lack of physical activity, among others, which can be improved through information given by teachers. Raising cancer awareness among adolescents has the potential to improve knowledge and confidence in identifying early symptoms and assisting in seeking early treatment.^7^ Several studies have demonstrated the importance of these actions in changing behavior among adolescents, including in relation to friends and family.^8^, ^9^ This information offered to students in a repeated and dynamic way, enables students to build knowledge, have critical thinking, and retain knowledge for life on the topic.^10^ Head and Neck Surgery is a specialty that was recognized worldwide in 1957. It mainly treats benign and malignant tumors of the face, nasal cavities, paranasal sinuses, mouth, pharynx, larynx, thyroid, salivary glands, and soft tissues. neck, parathyroid, and skin in the region. The area of expertise of the head and neck surgeon does not cover tumors or diseases of the brain and other areas of the central nervous system, nor those of the cervical spine.^11^ There is a certain lack of knowledge and confusion among the population about tumors in these locations, which may be caused by the fact that the anatomical regions interface with other specialties.^11^

Head and neck cancer account for about 4% of all cancers in the United States.^12^ According to INCA (National Cancer Institute), in 2020, about 65,630 people (48,200 men and 17,430 women) developed head and neck cancer.^13^ With the exception of non-melanoma skin cancer, which is the most frequent tumor, oral cavity cancer (among men) and thyroid cancer (among women) are the most frequent in Brazil.^13^ The risk factors associated with head and neck cancer include tobacco use and alcohol abuse. In addition, Human Papillomavirus (HPV) infection, acquired mainly through unprotected sexual intercourse, and Epstein-Barr virus (EBV) are responsible for part of oropharyngeal and rhinopharyngeal tumors, respectively.^12^, ^13^

The aim of this study was to assess the level of knowledge among a group of adolescents about tumors in the head and neck region and their risk factors. Suggestions to improve learning on the subject were also evaluated, in order to better promote this knowledge and result in reliable information propagators, allowing greater attention to the prevention and diagnosis of diseases in their early stages.

## Methods

The present study was conducted with 200 adolescents, anonymously, aged between 13 and 22 years-old, living in Recife, Pernambuco, located in the Northeast of Brazil, between November 11, 2020, and February 1, 2021, during the COVID-19 pandemic, where students were in online classes. 184 students, who responded to all questions, were included in the study. The exclusion criteria were questionnaires with more than three blank responses. As this study was about opinions on this topic with unidentified students, and we just used socio-demographic data and their own opinions, there is no need for the approval of the ethics committee in research.

The questionnaire was prepared by the researchers and administered to the students using google forms, sent by WhatsApp social media (See Appendix A – Questionnaire applied). We divided the fifteen questions into five sections: in the first section, we included sociodemographic features of the participants; the second section contained questions measuring the level of knowledge about cancer and family history; the third section has questions about head and neck cancer (sites, etiologic factors, and treatments); in the fourth section, we asked about adolescents perception about teaching oncological diseases and, finally, in the last section, we made an open question about suggestions to improve teaching on head and neck cancer.

### Data analysis

The variables were described by absolute and relative frequencies and by average, standard deviation, and minimum and maximum values. For data tabulation and analysis, the Microsoft Excel® program (Microsoft Corporation®, Redmond, Washington, USA) was used.

The qualitative evaluation was performed by analysis of repetitions according to the methodology proposed by Minayo, which aimed to critically review the theories on the subject in a specific and contextualized way.^14^ This analysis was extracted from the narratives of the students. In the case of qualitative research, the relevance considered was the repetition of information by the students in the sample obtained and not the size of the sample itself.

## Results

Of the 184 study participants, 63.6% (117) were women. The mean age of the participants was 15.8 ± 1.69 years (minimum of 13 years and maximum of 22 years).

All participants stated that they knew what cancer was. Regarding the questioning of cancer cases in the family, 123 (66.8%) interviewed said they had cases of cancer in the family, 47 (25.5%) denied cases and 14 (7.6%) were unaware of the existence of the disease in your family members.

**Fig. 1:**
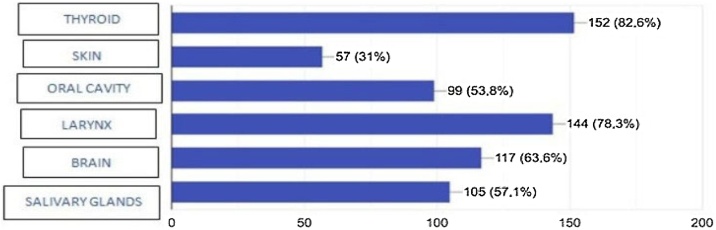
Question 5-what regions are treated by head and neck surgeon?

In the question regarding the regions treated by head and neck surgery, 152 (82.6%) knew that the thyroid is one of the anatomical sites treated by the specialty, followed by the larynx with 78.3% (144 responses), the brain in 63.6% (117), the salivary glands with 57.1% (105), the mouth with 53.8% (99) and the skin with 31% (57) (Fig. 1). One hundred and thirty-seven students (74.5%) denied not knowing the areas of the previous question, while 47 (25.5%) said they did not know any of these anatomical regions, and 15 (31.9%) students reported that they did not know the thyroid and eight (17%) students did not know the larynx.

**Fig. 2:**
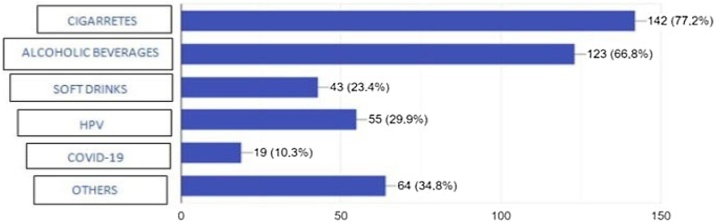
Question 8-what factors are involvedin etiology of head and neck cancer?

As for the etiological factors related to head and neck cancer, smoking was identified as a factor related to the development of cancer in this region by 142 students (77.2%), followed by alcohol consumption (123 students; 66.8%). Fifty-five students (29.9%) identified HPV as an etiological factor, while COVID-19 was reported by 19 students (10.3%). The use of soft drinks was mentioned by 43 students (23.4%). Other factors were mentioned by 39 (21.2%) of the interviewed, with 7 (18%) indicating more than one factor, including: genetics (18; 46.15%), sun exposure (9; 23.07%), drugs (6; 15.38%), food (5; 12.82%), radiation (5; 12.82%) and lack of physical activity (2; 5.12%). Industrialized products, hours of sleep, hormone levels, obesity, lip trauma, “not knowing how to take care of yourself”, stress, and cell phone use were also cited, each with 1.2%, as etiological factors for the development of head cancer and neck (Fig. 2).

Sixty-seven (36.4%) students knew how the treatment of oncological diseases was in the head and neck region, while 117 (63.6%) respondents were unaware of the appropriate treatment.

Regarding the question about which treatment is considered effective, six students (3.3%) reported radiotherapy, 20 (10.9%) mentioned chemotherapy, 27 (14.7%) mentioned surgery, and 131 (71, 2%) believed that all associated treatments worked on head and neck cancer.

About the definition what is radiotherapy and chemotherapy, 110 (59.8%) and 175 (95.1%) of the students interviewed, respectively, knew what these treatments were.

**Fig. 3:**
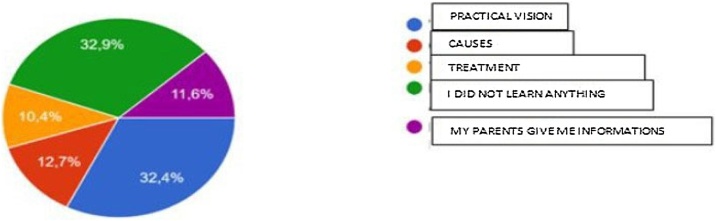
Question 14-what do you miss from being taught in classroom about oncological diseases?

In the field of learning about this topic during high school, 56 (32.4%) students reported having a deficiency in practical vision, 57 (32.9%) mentioned that did not learn anything about this subject in the classroom, 22 (12.7%) mentioned the lack of approach to the etiological agents, while 18 (10.4%) reported did not receive information about the treatment. Interestingly, 20 (11.6%) students reported that they received information about head and neck cancer from their parents (Fig. 3).

## Qualitative results

The open question asked about suggestions regarding the teaching of head and neck cancer in high school. 90 (48.9%) responses were obtained with multiple suggestions. These suggestions range from practical classes in specialized hospitals and classes given by professionals in the field, with a focus on signs and symptoms and, mainly, on prevention, with some cancer foci should be highlighted: (1) “These diseases need to be taught in schools in terms of prevention.”; (2) ‘These diseases need to be taught in schools in prevention.”; (3) “Practical classes in prevention” with hospitals or clinics, as well as teaching on emotional and personal memory stimulation with cancer cases.”; (4) “Take students to medical centers and, with that, explain the main disease in that environment, dictating causes, care, and treatments.”; (5) “Classes with specialists in the subject!”. There was a recurring complaint among the interviewees about the teaching deficit, referred to as (1) “Topics that are important in life are not taught in school”; (2) “I think this topic is one of the important topics that should be taught in college, but they don’t.”; (3) “Not always the vision of how this treatment is shown in classrooms, that is, not everything is said in the book.”

Another very interesting observation from the interviewees refers to the demystification and popularization of the subject: (1) “It has to be treated more normally, there is little information passed on and very superficial.” (2) “Schools could address these issues as they are extremely necessary for life.”; (3) “It should be a mandatory part of biology because it is a public utility”; (4) “A broader study of the different types of cancer, their causes, and signs, mainly in order to help someone ‘layman’ identify such signs and seek help.”

In addition to the complaints, the students also made suggestions about the dynamics and methodology of teaching, in addition to emphasizing that this teaching should be made available in biology classes: (1) “It could be more addressed in biology.”; (2) “Inform more about the types of cancer in Podcasts, video classes…” (3) “It should have informative talks followed by Q&A [from English: questions and answers].”; (4) “Divide the room into groups and each one would be responsible for studying/presenting about a certain disease and/or showing reports of people who have these diseases and how they discovered it. That way you never forget what you learned, and you will be much more informed”; (5) “Video class. It is a practical way where you will have a greater reach of people interested in the subject.”

## Discussion

This study on knowledge about head and neck cancer exclusively among adolescents in the state of Pernambuco is the first to be carried out in our country. It showed that knowledge is limited and there is a high degree of disappointment among students about what is taught in the classroom on the topic. Intervening effectively and early in order to promote health is essential for the prevention of diseases, including head and neck cancer, a preventable disease in most patients, with the adoption of healthy lifestyle habits.

The definition of adolescence, which dates back to the mid-twentieth century, when growth patterns and the timing of transitions were different and varied in most different parts of the world, can be considered backward. According to recent studies, the expansion of this range to 10–24 years old can be considered more in line with reality.^1^, ^15^ This expansion of the age group of adolescence is important because it is during this period that various concepts and knowledge are sedimented, leading to a change in habits. From the knowledge acquired in this age group, the propagation of information inside and outside the family nucleus is boosted, leading everyone to incorporate healthier and prevention habits. Knowledge also helps in the suspicion and identification of initial lesions in head and neck, leading to an early diagnosis with better results in treatment and survival, as also evidenced in the study by Kanmodi et al.,^16^ on teaching about head and neck cancer among schoolchildren. Finally, the chain of knowledge of these young people, guided by high school teachers and by themselves, can really change the reality of advanced diagnoses. This “oncological universe” is still a very unknown area for this age group. There are few published articles about the level of knowledge about cancer in this age group, and head and neck cancer, in general, has not been the object of much evaluation, being found in the literature mainly studies on cancer of the oral cavity and skin.^17^, ^18^, ^19^, ^20^, ^21^, ^22^

Regarding knowledge about the areas treated by head and neck surgery, 82.6% identified the thyroid, 78.3% mentioned the larynx, and 53.8% the mouth, however, 63.6% identified the brain as an area treated by head and neck surgeons, which is a mistaken notion among the population that Head and Neck Surgery deals with neoplasms in the brain, for example, perpetuating the lack of information about the limits with other specialties, such as Neurosurgery. Another important finding is that 25.5% reported not knowing any of the anatomical regions addressed in the study, with the thyroid (31.9%) and larynx (17%) being the most unknown organs, which perhaps also represents a failure in anatomical teaching among these young people. This observation is corroborated by the study by Carvacho et al.,^23^ it also refers to the lack of anatomical knowledge among adolescents. The authors concluded, for example, that there is a low level of knowledge about the anatomy of the reproductive system among pregnant adolescents.

In our study, 77.2% and 66.8% of students identified, respectively, smoking and alcoholism as risk factors, while 29.9% identified HPV as a triggering factor for head and neck cancer and 10.3% identified COVID-19 infection as a cancer-related factor in this region. The sun exposition was identified in 23.1% and only 1.2% trauma as risk factors. These results bring us an alert that knowledge about relevant risk factors is not of a broad domain in this age group, consistent with previous studies that also identified a lack of knowledge of risk factors.^17^, ^18^, ^19^, ^20^, ^21^, ^22^, ^24^

Most students (63.6%) did not know the appropriate treatment for head and neck cancer, while 3.3% reported radiotherapy as efficient treatment. About conceptualization, however, 59.8% did not know what radiotherapy is and only 4.9% did not know about chemotherapy. The expressive understanding of what chemotherapy is by these students can be justified by the high incidence of breast cancer in the state of Pernambuco (2020–2022 estimate of 2,390 cases),^13^ associated with the question we asked if there were cases of cancer in the family, which correspond to at 66.8%. In this context, perhaps the family experience justifies these findings.

Even though head and neck cancer is a very specialized topic, we believe that it is important for students to be guided about the treatments in addition to the effectiveness of each one, so that the stigma that the diagnosis of the disease has a harmful meaning is removed. When knowledge becomes broader, the population will begin to seek care and start treatment as early as possible.

In the evaluation of the open response, the suggestions were diversified, but always with a focus on prevention guidance and early detection of signs and symptoms, in addition to demystifying the subject. Students complain a lot about classes on specific subjects which, when they exist, generally have only theoretical focus and lack the practical part. Regarding learning about head and neck neoplasms and their treatments, 83.1% of the students made some type of complaint about knowledge that is not transmitted, either about risk factors or about treatment. 32.9% of these students reported still not learning anything about this topic. Abraham et al.^24^ reported in their study on the perception of adolescents about cancer and education preferences, the importance of developing strategies for teaching the topic among this age group, and how much the change in this teaching pattern can impact the level of knowledge and disease control, which is in line with the suggestions of the interviewees in this study (talking to specialists, video classes, practical classes, among others).

Finally, a very striking observation, said by the students themselves, has led to many discussions among specialists and several suggestions for changes in secondary education: “Topics that are important in life are not taught in school”. This concern about teaching methodologies led to the development of a pilot project, developed by the University of Porto, called “Cancer – Educating to prevent”. In this project, secondary school biology teachers receive training focused on the five most common tumors in Portugal (colorectal, stomach, breast, cervix, and skin), allowing for efficient training of educators and an impact on the students’ knowledge level.^25^

Our study had some limitations. The number of students evaluated is small and of a wide age group (between 13 and 22 years-old). Another important point to be mentioned is that adolescents who answered the questionnaire belonged to a higher socioeconomic level than a large part of the Brazilian population. In addition, these students were recruited exclusively from private education and also from a specific region of the country. This point, however, is not understood by the authors as a weakness, but rather a point of even greater attention regarding the relevance of our findings. The study identified a significant lack of knowledge on the subject, even in a group with a high socioeconomic level, that is likely that Brazilian underprivileged adolescents, which represents about 75% of this population, have even more prejudice against this important information. Health literacy is essential to achieve better results in the prevention and treatment of various diseases, including cancer.

## Conclusion

We conclude that knowledge about head and neck cancer is deficient among adolescents and there is a need to change teaching so that they can introduce healthy attitudes into their lives, and those around them, and also be able to detect signs and symptoms of cancer in head and neck earlier. It is also evident that students need more knowledge, and they would like more practical classes and “in the field” for better learning on the subject. Certainly, if a large part of the students learned in the school environment, guided by teachers, who are often admired and are opinion formers, or health professionals, we would have a “pay it forward”, with a change of several paradigms. Only knowledge will lead to a change in attitude and the sooner these lessons are acquired, the better will be the health of the entire population.

## Declaration of competing interest

The authors declare no conflicts of interest.

## Figures and Tables

**Table:** 

Knowledge about head and neck cancer among adolescents
1. Age
2. Gender
( ) Female
( ) Male
( ) Other
3. Do you know what cancer is?
( ) Yes
( ) No
4. Is there any case of cancer in your family?
( ) Yes
( ) No
( ) I do not know
5. What regions below are treated by head and neck surgery?
( ) Thyroid
( ) Skin
( ) Mouth
( ) Larynx
( ) Brain
( ) Salivary glands
Are any of the anatomical regions described in the above question unknown to you?
( ) Yes
( )No
6. What regions do you not know?
7. What factors below are involved in the etiology of head and neck cancer?
( ) Cigarettes
( ) Alcoholic beverages
( ) Soda
( ) HPV
( ) COVID-19
( ) Other
8. What are other factors?
9. Do you have any idea how to treat oncological diseases in the head and neck region?
( ) Yes
( ) No
10. What treatment do you believe is effective?
( ) Surgery
( ) Radiotherapy
( ) Chemotherapy
( ) All above
11. Do you know what radiotherapy is?
( ) Yes
( ) No
12. Do you know what chemotherapy is?
( ) Yes
( ) No
13. What do you miss from being taught in classroom about oncological diseases?
( ) Practical classes
( ) Causes
( ) Treatment
( ) I learn nothing about this subject in the classroom
( ) My parents give me this information
14. Leave your suggestions about how to teach these diseases.
